# Multidisciplinary Perioperative Care for Children with Neuromuscular Disorders

**DOI:** 10.3390/children5090126

**Published:** 2018-09-12

**Authors:** J. Matthew Kynes, Martin Blakely, Kevin Furman, William B. Burnette, Katharina B. Modes

**Affiliations:** 1 Monroe Carell, Jr Children’s Hospital at Vanderbilt, Nashville, TN 37232, USA; j.matt.kynes@vumc.org (J.M.K.); martin.blakely@vumc.org (M.B.); william.b.burnette@vumc.org (W.B.B.); 2 Vanderbilt University Medical Center, Nashville, TN 37232, USA; kevin.furman@vumc.org

**Keywords:** neuromuscular disorder, mitochondrial disease, muscular dystrophy, rhabdomyolysis, malignant hyperthermia, anesthesia, perioperative care

## Abstract

Children with neuromuscular diseases present unique challenges to providing safe and appropriate perioperative care. Given the spectrum of disease etiologies and manifestations, this is a population that often requires specialized multidisciplinary care from pediatricians, geneticists, neurologists, dieticians, and pulmonologists which must also be coordinated with surgeons and anesthesiologists when these patients present for surgery. Several of these diseases also have specific pharmacologic implications for anesthesia, most notably mitochondrial disease and muscular dystrophies, which put them at additional risk during the perioperative period particularly in patients presenting without a formal diagnosis. Techniques and strategies to fully evaluate and optimize these patients preoperatively, manage them safely intraoperatively, and return them to their baseline status postoperative are particularly important in this vulnerable group of patients. Utilizing a review of inherited neuromuscular conditions, generalized perioperative concerns, and specific complications related to anesthesia, this article provides an overview of pertinent considerations and recommends a framework for management of these patients.

## 1. Introduction

Neuromuscular disorders (NMDs) represent a range of heterogenous conditions diagnosed in children presenting with myopathy, motor delay, seizures, and other manifestations of abnormalities in nerve conduction and muscle contraction. Although individual disorders may be rare, when the prevalence of conditions resulting in abnormal motor function is combined their numbers grow substantially. The combined rate of NMDs has been estimated at 160/100,000 [1], which is twice as high as multiple sclerosis and similar to Parkinson’s disease. Given the complexity and costs associated with managing these chronic conditions, a working knowledge of underlying mechanisms and clinical considerations for NMDs and coordination of care between pediatricians, neurologists, geneticists, and therapists caring for these patients is important. Patients with NMDs commonly undergo surgery for diagnosis and management of symptoms and complications of their disease which requires additional coordination among pathologists, surgeons, and anesthesiologists to optimize their care. In light of the unique interaction between anesthetic agents and certain NMDs, this review will focus on perioperative considerations for muscular dystrophies, mitochondrial disease and undiagnosed myopathic disorders and propose considerations to aid providers caring for this vulnerable population. While other NMDs require perioperative coordination of care, such as cerebral palsy and other static or progressive secondary NMDs, the focus of this review is conditions with unique anesthetic considerations with the understanding that many principles will be applicable to a range of neuromuscular conditions.

## 2. Neuromuscular Disorders in Children

A complete list of NMDs includes more than seventy diagnoses and, while multiple classification schemes exist, they can be broadly divided into muscular dystrophies, motor neuron diseases, ion channel diseases, mitochondrial diseases, myopathies, neuromuscular junction diseases, and peripheral nerve diseases. These may have genetic or nongenetic causes and may present in infancy, childhood, or adulthood depending on the disease. Classification of NMDs has been challenged by progress in molecular genetics and syndromes have a wide overlap in phenotype even with similar or identical gene mutations. Diagnosis of NMDs may be based on clinical signs, chromosomal analysis, skin biopsy, or body fluid analysis and occasionally muscle biopsy under anesthesia is required.

Manifestations of NMDs result from abnormal motor function causing chronic conditions including ataxia, joint contracture, scoliosis and other skeletal deformity, systemic weakness, metabolic derangements, respiratory failure, cardiomyopathy, arrhythmia, and premature death. Symptoms may be constant, intermittent or progressive and long-term management is complex and costly. Depending on the severity of the disease, medical costs may include medications and interventions for disease and symptom management, frequent outpatient clinic visits, respiratory support including home ventilation, continuous tube feeding, frequent and prolonged hospitalizations. Nonmedical costs include loss of income to families, loss of opportunities for education, travel and housing accommodations necessary for frequent specialist care, and others. A 2013 estimate of annual per-patient costs for three neuromuscular conditions in adults ranged from $32,236 to $63,693 with total US costs of $1.07 to $1.37 billion [2], and inclusion of other diagnoses and consideration for the total duration of management for these chronic conditions put the costs for all NMDs present in children much higher.

### 2.1. Muscular Dystrophies

Muscular dystrophies (Table 1) are an inherited group of disorders that are characterized by progressive wasting of skeletal muscles. These diseases include Duchenne muscular dystrophy (DMD), Becker, Emery–Dreifuss, limb-girdle, and myotonic dystrophies. The hallmark of the muscular dystrophies are genetic defects leading to altered muscle protein development, particularly the dystrophin–glycoprotein complex, and progressive muscle degeneration. Of particular concern in muscular dystrophy is the possibility of cardiac involvement, present in at least 20% [2]. Indeed, the absence of significant skeletal muscle symptoms does not preclude the possibility of severe cardiac conduction or contraction abnormalities. DMD, due to an X-linked recessive mutation, is the most common form and occurs in 1 in 3500 boys, leading to death from respiratory or cardiac failure before or during the fourth decade of life [3].

### 2.2. Mitochondrial Disease

Mitochondrial disorders (Table 1) involve defects in electron chain transport or oxidative phosphorylation that manifest systemically in organ systems with high energy requirements [1] and affect 1 per 4000 live births [5]. Mutations causing mitochondrial myopathies may occur in mitochondrial or nuclear DNA. The brain, heart, and muscle are particularly vulnerable to mitochondrial defects and, while mitochondrial disorders are phenotypically heterogenous, they may present with hypotonia, central nervous system (CNS) dysfunction, cardiomyopathy, respiratory insufficiency, and metabolic acidosis. Named syndromes associated with symptom clusters include Kearns–Sayre syndrome, mitochondrial encephalomyopathy with lactic acidosis and stroke-like episodes (MELAS), and Leigh syndrome, although due to the wide range of defects and presentations many patients do not fit into one particular grouping.

## 3. Perioperative Complications in Patients with Neuromuscular Disorders

### 3.1. General Perioperative Complications

#### 3.1.1. Respiratory Muscle Weakness

In patients with moderate-to-severe forms of NMDs, weakness of the diaphragm and accessory respiratory muscles can combine with spinal deformity, dysphagia, and recurrent aspiration to cause significant respiratory compromise. Some disorders are more likely to present with acute respiratory failure, while others manifest with chronic respiratory failure that may be rapidly, slowly or variably progressive [6]. In DMD, vital capacity declines by 8% annually after onset around 2–5 years of age and nocturnal noninvasive ventilation is usually required after age 15 [7]. In many patients, sleep-disordered breathing is the first manifestation of respiratory muscle weakness which can progress to rapid respiratory muscle fatigue, mucous plugging, pneumonia, atelectasis, and respiratory failure. Multiple volitional and nonvolitional tests of respiratory muscle function are available to assess the degree of impairment including maximum inspiratory and expiratory pressure, peak cough flow, sniff nasal inspiratory pressure, phrenic nerve conduction, ultrasound assessment of diaphragm thickness, and others. Diaphragmatic weakness, for instance, can be predicted by a more than 20% drop in vital capacity from upright to supine position [8], although the validity, reliability, and availability of this and other measures are limited [6]. Steroid therapy, noninvasive and invasive ventilation including tracheostomy, secretion management, and respiratory muscle training are interventions commonly utilized to manage progressive respiratory muscle weakness and its sequelae.

#### 3.1.2. Cardiomyopathy and Conduction Abnormalities

While the majority of NMDs result in defects within striated muscle through derangements in nerve transmission, mitochondria, ion channels or muscle proteins resulting in progressive weakness, many also have an effect on cardiac myocytes. Cardiac involvement includes effects on contractility-producing cardiomyopathy or on conduction-producing arrhythmia. Manifestation of cardiac symptoms commonly occurs in childhood or adolescence, although some forms of NMD remain asymptomatic until late in life. Interestingly, progression of skeletal muscle and cardiac dysfunction correlate poorly. In DMD, cardiac disease produces decreased ventricular ejection by age 14 years and significantly shortens survival [9]. Due to the limited activity imposed by progressive weakness, diagnosis of cardiac abnormalities may be significantly delayed and genetic diagnosis to predict degree and type of dysfunction and regular screening for cardiac involvement are critical components in management [10]. As cardiac disease progresses in patients with NMDs, inhibition of the renin-angiotensin-system, beta blockade, antiarrhythmic treatment, pacemaker or implantable cardiac defibrillation may all become necessary.

### 3.2. Disease-Specific Perioperative Complications

#### 3.2.1. Malignant Hyperthermia Susceptibility

Malignant hyperthermia (MH) is a pharmacogenetic disorder that complicates one in 30,000 surgeries in children and causes unopposed skeletal muscle contraction after exposure to depolarizing neuromuscular blockers (i.e., succinylcholine) or volatile anesthetics (e.g., halothane, isoflurane, sevoflurane, desflurane) resulting in muscle rigidity, rhabdomyolysis, acidosis, arrhythmia, hyperkalemia, hyperthermia, and death. Sustained muscle contraction occurs from uncontrolled Ca^2+^ release from the sarcoplasmic reticulum and an inability of normal cellular mechanisms to restore myoplasmic homeostasis. Defects in the ryanodine receptor (RyR1) are primarily responsible for altered Ca^2+^ migration, although a number of proteins are involved and multiple forms of MH susceptibility have been identified with one form related to an altered RyR1-activating protein (CACNA1S) and another related to *STAC3* gene variants [11]. Despite recent advances in genetic testing, a complete understanding of a genetic basis for MH-susceptibility remains elusive and up to 50% of patients at risk of MH do not carry known MH-associated genes [12].

In the past, there has been some confusion regarding the risk of MH in patients with neuromuscular disease. Ryanodine receptor variants are the most common cause of nondystrophic congenital muscle disease [13] and may be a feature of several congenital myopathies including central core disease, multiminicore disease and King–Denborough syndrome. All of these have been directly linked with MH [11,12,13,14], although heterogeneity in RyR1 involvement and MH risk does exist (Table 3). In fact, most patients who possess RyR1 variants and have experienced MH have no clinical features of disease [13]. Overlap in clinical and histopathological features of these conditions and others, such as nemaline myopathy, and ongoing investigation and reclassification of NMDs according to genotype has generated additional debate regarding MH risk. In addition, many reported cases of MH are likely not true MH but rather episodes of anesthesia-induced rhabdomyolysis, which is understandable given the overlap in their presentation. As recent reviews have indicated [12,13], our understanding of MH susceptibility continues to evolve. For the purposes of this review, no muscular dystrophies or mitochondrial myopathies have known clinical or genotypic MH associations. It may be considered for patients with or having immediate family members with the genetic abnormalities listed. For symptomatic patients without a definitive genetic or clinical diagnosis of a specific neuromuscular condition, a large study of children undergoing muscle biopsy established the risk of MH at less than 1% [15]. A summary of disorders with MH-associated variants is provided in Table 2. 

#### 3.2.2. Anesthesia-Induced Rhabdomyolysis

As previously mentioned, patients with muscular dystrophies have an underlying defect in muscle protein development that predisposes them to progressive muscle degeneration. Muscle membrane stability—especially in DMD—may be further compromised by exposure to halogenated volatile anesthetic agents or succinylcholine, and cases of anesthesia-induced rhabdomyolysis (AIR) causing sudden cardiac arrest from hyperkalemia have been reported [14]. While the exact mechanism for AIR remains to be discovered, and presence of a muscular dystrophy and exposure to halogenated agents or succinylcholine does not cause rhabdomyolysis in every case, many practitioners advocate for the avoidance of these agents in the presence of known muscular dystrophy.

#### 3.2.3. Metabolic Abnormalities

Unlike other categories of NMDs which manifest through direct effects on muscle or nerve structure and function, mitochondrial disorders cause neuromuscular disease through defects in cellular metabolism which put patients at particular risk for metabolic decompensation [16]. Patients with mitochondrial myopathies are particularly reliant on glucose for cellular respiration and often present with elevated lactic acid levels from anaerobic metabolism in the setting of limited ATP reserve and impaired fatty acid metabolism. This limited energy reserve also puts these patients at risk for increased metabolic stress during periods of decreased energy supply (i.e., prolonged fasting, postoperative nausea, hypovolemia) or increased demand (hypothermia with shivering, pain, tachycardia, tachypnea) common in the perioperative period.

#### 3.2.4. Propofol Infusion Syndrome

While a variety of anesthetic approaches have been used safely for patients with mitochondrial myopathies [17,18] and past concerns about an association with MH have been dismissed [19], every anesthetic agent studied has been shown to depress mitochondrial function [20,21]. Desflurane, isoflurane, and sevoflurane suppress cellular respiration at complex I, V, and coenzyme Q. This has been well tolerated when depth of the anesthetic has been carefully monitored. The discontinuation of these volatile gases allows the mitochondria to return to normal function fairly quickly due to the rapid elimination and little metabolism they undergo. However, patients with disorders of fatty acid oxidation and mitochondrial respiratory chain function can develop significant complications with propofol, an anesthetic agent emulsified with a long-chain fatty acid carrier [22]. Rather than having effects on isolated complexes of the electron transport chain like other parenteral anesthetics, propofol inhibits complexes I, II, IV and inhibits acylcarnitine transferase, which inhibits the transport of acylcarnitine esters into the mitochondrion [23]. Although propofol infusions have been used safely in patients with mitochondrial disease, an association between these disorders and susceptibility to propofol infusion syndrome, which manifests as lactic acidosis, rhabdomyolysis, and lipidemia leading to cardiovascular collapse, has been proposed [24]. General and disease-specific perioperative considerations are summarized in Table 3. 

## 4. Multidisciplinary Considerations for Perioperative Care for Patients with Neuromuscular Disorders

Given the complexity of perioperative management in patients with NMDs and the potential for poor outcomes in patients that are not managed judiciously, there exists a need for establishing multidisciplinary guidance for standardizing the care these patients receive. A large retrospective study of patients with NMDs undergoing muscle biopsies revealed no serious complications, and its authors concluded that “increased awareness of anesthesia-related concerns in an NMD patient directs management along specific safe pathways” [25]. While increased awareness is evident from multiple existing reviews of perioperative concerns for patients with NMDs [5,17,18,19,20,24,26,27,28,29], most have been directed toward anesthesia providers and very few define in precise and actionable ways the “specific safe pathways” that should be followed throughout the perioperative continuum of care. Indeed, utilization of standardized guidelines to assist in management of these patients remains low. A 2013 survey of US anesthesia providers, for instance, revealed that 93% managed patients with mitochondrial disease in their practice but only 11% had institutional guidelines in place [24].

**Figure 1:**
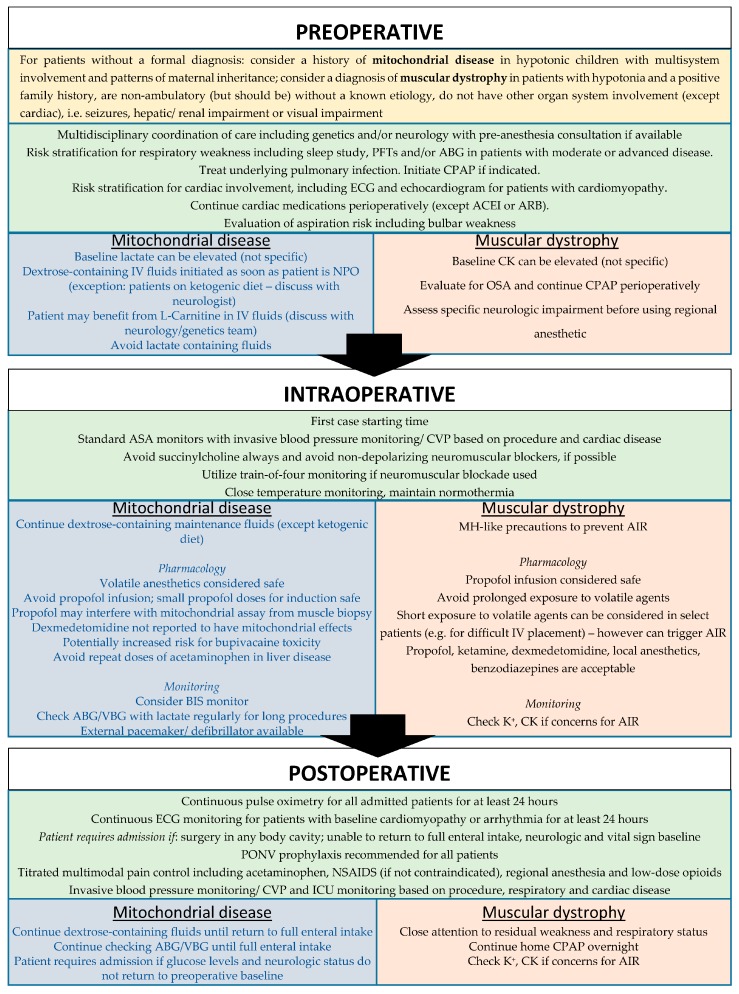
Perioperative framework for management of patients with neuromuscular disorders with specific considerations for mitochondrial disease and muscular dystrophies. PFT: pulmonary function test, ABG: arterial blood gas, CPAP: continuous positive airway pressure, ECG: electrocardiogram, ACEI: angiotensin-converting enzyme inhibitor, ARB: angiotensin II receptor blocker, NPO: *nil per os*; OSA: obstructive sleep apnea; CVP: central venous pressure; AIR: anesthesia-induced rhabdomyolysis, BIS: Bispectral index, PONV: post-operative nausea and vomiting, ICU: intensive care unit, VBG: venous blood gas, NSAIDS: non-steroidal anti-inflammatory drugs.

The following sections outline key considerations for preoperative workup, intraoperative management and postoperative monitoring of patients with NMDs in general, as well as mitochondrial disease and muscular dystrophies in particular. These considerations were formulated in consultation with available evidence in the literature as well as input from geneticists, pathologists, neurologists, and surgeons at our institution. Due to the paucity of prospective studies on perioperative management for patients with NMDs and the evolving understanding of these conditions, the goal of this section is to provide an organized framework for approaching patient management and not standard of care (Figure 1).

### 4.1. Preoperative Evaluation and Optimization

As described above, neuromuscular diseases have manifestations in multiple body systems and problems related to pulmonary and cardiac function are likely to cause the greatest harm in the perioperative period and should be assessed appropriate to the invasiveness of the procedure whenever possible. In many settings, perioperative planning may be coordinated by a preoperative anesthesia consultation from the surgical service ideally with ample time to gather additional information and input prior to surgery. If available, overnight oximetry or sleep studies should be reviewed to predict respiratory performance in a sedated state and may affect choice of anesthetic and analgesic approach and need for postoperative monitoring. Pulmonary function testing may reveal significant restrictive disease but a careful clinical assessment is of similar utility in predicting postoperative risk [30]. Pulmonary optimization includes treating underlying infection (particularly in the presence of chronic aspiration), initiating non-invasive ventilation for overnight support and managing airway hyperreactivity with inhaled steroid and beta-agonists as indicated. Training with mechanical insufflator–exsufflator (MI-E) and cough techniques in coordination with respiratory therapists has been advocated for pulmonary optimization, as well [26]. For patients dependent on continuous positive airway pressure (CPAP), bilevel positive airway pressure (BiPAP) or ventilator support via tracheostomy, current settings should be reviewed, and the patient or the patient’s family should be instructed to bring his or her home equipment on the day of surgery.

A basic evaluation of cardiac function including history of cardiac events, arrhythmias, and cardiomyopathy should be performed in all patients with NMDs, bearing in mind that the relationship between cardiac and skeletal muscle involvement is unpredictable. Screening is also required because patients with advanced NMD have limited physiologic stress from activity to reveal symptomatic cardiac disease. Preoperative screening for patients undergoing muscle biopsy for NMD diagnosis can reveal ventricular dysfunction in up to 17%, and a combination of electrocardiography (ECG) and chest X-ray may be predictive of left ventricle (LV) dysfunction in 81% of patients [27]. Echocardiography may be utilized in selected patients and is particularly useful to distinguish between moderate and severe dysfunction, especially if not available in the previous 12 months [26]. Cardiac medications, with the possible exception of angiotensin-converting enzyme inhibitors and angiotensin receptor blockers, should be continued preoperatively.

Assessment of the patient’s baseline neurologic status will help gauge the level of cooperation to be expected and inform management of any changes occurring postoperatively. Current seizure management including anti-epileptic drugs and dietary changes, such as the ketogenic diet, should be maintained throughout the perioperative period including the day of surgery. Patients with poorly controlled seizures may require referral for medication adjustment prior to elective surgery. Other recommended optimization considerations include nutritional supplementation and a complete discussion of risks and benefits of surgical and anesthetics risks for a given procedure [26].

#### 4.1.1. Preoperative Evaluation and Optimization for Muscular Dystrophies

Cardiac involvement may be particularly problematic in patients with muscular dystrophies and additional evaluation may be warranted. A transthoracic echocardiogram to evaluate LV function or a combination of ECG and chest X-ray should be considered to rule out cardiac involvement. Recent cardiac workup of patients with known cardiac disease should be reviewed along with any specific recommendations from the patient’s treating cardiologist. Other specific preoperative evaluation should include creatinine kinase, which is often significantly elevated in muscular dystrophy. Obtaining a baseline value preoperatively can be helpful in assessing intraoperative and postoperative changes.

#### 4.1.2. Preoperative Evaluation and Optimization for Mitochondrial Diseases

In addition to the general approach to patients with NMDs described above, patients with mitochondrial disorders have other special considerations. Detailed information regarding the patient’s specific disorder, functional status and current medication regimen should be obtained from the treating geneticist or neurologist. Baseline laboratory values including glucose, lactate level, liver function tests, and basic metabolic panel should be obtained prior to surgery if recent values are not available. Lactate levels are particularly relevant as values are often elevated in mitochondrial disorders and a trend intraoperatively and postoperatively can guide therapy.

Cardiac conduction defects or pre-excitation syndromes commonly occur in mitochondrial disorders and should be excluded by electrocardiogram preoperatively. Additionally, echocardiogram should be considered in disorders associated with hypertrophic cardiomyopathy such as MELAS or Leigh syndrome.

Procedures for patients with mitochondrial disorders should be scheduled early in the day and fasting times should be minimized since glucose stores are quickly depleted. Clear liquid intake should be encouraged up until two hours prior to arrival time. Common preoperative supplements include vitamins, coenzyme Q, and L-Carnitine and these should be continued on the day of surgery. Patients with expected poor tolerance even to short fasting times may require admission the night prior to their procedure to allow for initiation of maintenance IV fluids with a continuous glucose source [31]. The need for this should be determined in consultation with the patient’s neurologist or geneticist. One important caveat is that some patients with certain mitochondrial myopathies or seizures may be maintained on a ketogenic diet to stimulate mitochondrial biogenesis and decrease oxidative stress which precludes the administration of glucose-containing fluids [32]. Because of the possibility of elevated baseline lactate, lactate-containing fluids should be avoided.

### 4.2. Intraoperative Management

The utilization and avoidance of certain anesthetic agents for specific conditions is described below, but there are several intraoperative considerations common to all patients with NMDs. Succinylcholine should be avoided in patients with hypotonia or neuromuscular disease due to concerns for risk of MH, rhabdomyolysis, and hyperkalemia from upregulation of acetylcholine receptors [28]. Reports on the response of patients with mitochondrial disease to nondepolarizing muscle relaxants vary [33]. They should be used with caution and close train-of-four (TOF) monitoring is required (including establishing a TOF baseline before administration of neuromuscular blockade) with sugammadex reversal when available [34]. Where possible, muscle relaxation should be avoided entirely [26].

Intraoperative considerations for patients with cardiac involvement may include close monitoring of volume status, electrolyte repletion, ensuring immediate availability of cardiac pacing and defibrillation, and vasopressor administration.

There are several changes that occur in the perioperative period that can exacerbate respiratory compromise in patients with NMDs. Intraoperatively, endotracheal intubation and prolonged supine positioning impair clearance of secretions and lead to atelectasis, both of which are more difficult to reverse in patients with respiratory muscle weakness. Most anesthetic agents and all neuromuscular blocking drugs cause generalized muscle weakness which is unlikely to return to baseline in the immediate postoperative period [35] and can contribute to hypoventilation, atelectasis, and aspiration.

Nearly all anesthetic agents are direct cardiac depressants and have the potential to cause cardiovascular collapse in patients with limited cardiovascular reserve. Common physiologic changes in the perioperative period include tachycardia, hypovolemia, anemia, electrolyte disturbance and other fluid shifts which may not be tolerated in vulnerable patients.

#### 4.2.1. Intraoperative Management for Muscular Dystrophies

The first anesthetic exposure for patients suspected of suffering from muscular dystrophy often involves minimally invasive procedures such as imaging studies of the brain to evaluate hypotonia or muscle biopsy. Patients with known muscular dystrophy will frequently present for orthopedic procedures. As previously outlined, the main intraoperative concerns involve the risk of AIR and hyperkalemic cardiac arrest. Procedures should be scheduled early in the day, and MH-like precautions to eliminate halogenated volatile anesthetics from the operating environment should be strongly considered. Depending on the patient’s diagnosis and circumstances (e.g., history of difficult IV placement), short exposure to volatile anesthetics can be considered in select patients. However, this must be weighed against the risk of AIR and hyperkalemia. Total intravenous anesthesia is generally recommended, and some patients may have bulbar involvement and would benefit from a modified rapid sequence induction. Ketamine, dexmedetomidine, propofol, nitrous oxide, and local anesthetics are acceptable anesthetic agents for patients with muscular dystrophies. Opioids may be utilized for pain control but use must be weighed against the risk of respiratory depression.

#### 4.2.2. Intraoperative Management for Mitochondrial Diseases

Patients with mitochondrial disease present for procedures ranging from simple imaging studies to invasive surgical operations. Induction of anesthesia can be performed through an existing IV catheter or by inhaled induction. A known history or clinical suspicion for delayed gastric emptying may warrant modified rapid sequence induction.

Anesthetic selection for patients with mitochondrial disease is determined by several factors. Volatile anesthetics are generally considered safe, although increased sensitivity to sevoflurane has been reported for patients with mitochondrial disorders, especially involving complex I [23]. Application of a bispectral index (BIS) monitor can be helpful in guiding anesthetic depth. Even though an induction dose and small intermittent boluses of propofol are considered reasonable, long infusions of propofol are advised against because patients may be more susceptible to developing propofol-infusion syndrome, as described above. Benzodiazepines and ketamine are thought to be well tolerated. No reported mitochondrial effects have been found with dexmedetomidine, and its use has been widely accepted. Local anesthetics may be utilized for subcutaneous infiltration or nerve blockade; however, lidocaine and ropivacaine are preferred over bupivacaine. All inhibit the carnitine-acylcarnitine translocase, but lidocaine and ropivacaine do so to a lesser extent, allowing cellular respiration to continue [33].

Autonomic regulation can be impaired in mitochondrial disease which requires close attention to temperature control in the operating room and in the immediate postoperative period.

### 4.3. Postoperative Management

Respiratory and cardiac monitoring and support should be immediately available to all patients with NMDs in the immediate postoperative period and should include supplemental oxygen, continuous ECG monitoring, and pulse oximetry. Due to their respiratory depressant effects, opioids need to be titrated carefully and alternative means of providing postoperative analgesia should be utilized when possible including acetaminophen, ketamine, and nonsteroidal anti-inflammatory drugs. Regional and/or local anesthesia should be employed when available, as the opiate sparing effects of such techniques provide less respiratory inhibition [26,33].

Small, noninvasive procedures and diagnostic studies with general anesthesia can be considered on an outpatient basis if the patient’s neurologic status has returned to baseline and the patient is able to tolerate full enteral intake in the recovery room [29]. The patient’s caretakers need very clear instructions on what symptoms should prompt a return to the hospital. For more invasive procedures, intensive postoperative monitoring and cardiovascular support may also be necessary, particularly for patients with moderate-to-severe cardiac or respiratory dysfunction. Protocolized application of noninvasive ventilation and MI-E for high-risk patients has the potential to decrease reintubation and intensive care unit (ICU) length of stay [26].

#### 4.3.1. Postoperative Management for Muscular Dystrophies

As in other neuromuscular conditions, the duration of postoperative monitoring depends on the invasiveness of the procedure and the severity of the muscular dystrophy. Judicious pain management preferably with multimodal, opioid-sparing techniques in the presence of respiratory insufficiency are preferred.

#### 4.3.2. Postoperative Management for Mitochondrial Diseases

Given the propensity of patients with mitochondrial disorders to develop hypoglycemia, IV fluids should be continued and glucose as well as lactate levels should be checked at regular intervals until enteral intake is established. Postoperative nausea and vomiting should be treated aggressively with standard anti-emetic agents. Pain, hyperthermia, and hypothermia with shivering increase energy requirements and, if present, should be addressed promptly in the recovery room.

## 5. Perioperative Approach to Patients with Undiagnosed Neuromuscular Disorders

Many infants and children with unspecified neuromuscular disease are referred for diagnostic or surgical procedures requiring anesthesia as part of their workup. As outlined above, the risk of complications in symptomatic patients is elevated and anesthetic choice can have a profound effect on the perioperative course. In early stages of diagnosis, it is particularly difficult to determine the most likely etiology from the limited information available preoperatively. Even when a diagnosis is given preoperatively, up to 10% of patients undergoing muscle biopsy may carry a different diagnosis postoperatively [25].

Preoperative evaluation, intraoperative management, and postoperative management for patients without a specific diagnosis should be guided by considerations applicable to all patients with NMDs. During muscle biopsy, the risk of developing MH for a patient with an undefined NMD with exposure to volatile anesthetics is estimated to be 1.09% [15]. Some anesthetics such as ketamine, dexmedetomidine, and midazolam are considered good options both for patients with mitochondrial disease and muscular dystrophy. Opioid medications may cause respiratory depression and should be titrated carefully for pain control or avoided. Where anesthetic options differ between these conditions and no known safe alternative exists, the risks and benefits of a particular approach must be weighed and a course chosen based on the most likely underlying diagnosis in consultation with a multidisciplinary team whenever possible.

Clues to differentiate between mitochondrial disorders and muscular dystrophies include multisystem involvement being more consistent with mitochondrial disease, and a strong family history and lack of involvement of other organ systems (except cardiac) in the setting of muscle weakness suggesting muscular dystrophy to be the more likely etiology. Similarly, an elevated lactate level, although not very specific, is more frequently found in mitochondrial disease while an elevated creatinine kinase level would point towards muscular dystrophy. For patients undergoing muscle biopsy for diagnosis of a suspected mitochondrial disorder, propofol’s inhibition of complexes in the electron transport chain can theoretically lead to a false interpretation of a positive mitochondrial assay and should be avoided if alternatives are available [36].

## 6. Conclusions

Neuromuscular disorders present unique management challenges in the outpatient and perioperative settings. The severity of presentation and potential for complications vary for individual conditions and patients and an individualized approach coordinated among multiple providers is critical to optimizing outcomes for these vulnerable patients. A knowledge of certain guiding principles used to inform a standardized approach is also critical, particularly given the special anesthetic implications of certain disorders. As more information on the underlying genetic causes leads to a better understanding of disease manifestations and therapies, the management of children with these conditions will continue to evolve.

## Figures and Tables

**Table 1:** Overview of the most common muscular dystrophies and mitochondrial disorders with specific anesthetics implications and notable features for each [3,4].

Neuromuscular Disorder	Notable Features
**Muscular dystrophies (MD)**	*Muscle diseases with spectrum of severity including focal or diffuse weakness and cardiac dysfunction.*
Becker MD	Progressive weakness with calf hypertrophy with preservation of neck flexor strength; serum CK > 5× normal; average onset cardiomyopathy 14.6 years old. X-linked, altered dystrophin expression.
Duchenne MD	Progressive weakness with calf hypertrophy; serum CK > 10× normal; 50% with cardiomyopathy by age 18 years. X-linked, absent dystrophin expression.
Emery–Dreifuss MD	Joint contractures, progressive weakness, cardiac involvement; cardiac conduction defects and/or cardiomyopathy. *EMD*, *FHL1*, *LMNA* gene involvement.
Limb-girdle MD	Skeletal muscle involvement with variable progression. Multiple subtypes based on autosomal dominant or recessive traits.
**Mitochondrial disorders**	*Abnormalities of mitochondrial respiratory chain with heterogenous manifestations commonly including ophthalmoplegia, myopathy, cardiomyopathy, encephalopathy, seizures, and spasticity.*
Kearns–Sayre syndrome (KSS)	Pigmentary retinopathy and external ophthalmoplegia; elevated lactate; cardiac conduction abnormalities; ragged-red fibers on muscle biopsy. Mitochondrial DNA (mtDNA) deletion
Leigh syndrome	Hypotonia, movement disorders due to brainstem and/or basal ganglia involvement; hypertrophic cardiomyopathy; elevated lactate. mtDNA variant (*MT-ATP6* most common) or mtDNA deletion.
Mitochondrial encephalomyopathy, lactic acidosis and stroke-like episodes (MELAS)	Elevated lactate and seizures; ragged red fibers on muscle biopsy; hypertrophic cardiomyopathy; pigmentary retinopathy. mtDNA defect (*MT-TL1*).
Myoclonus epilepsy with ragged red fibers (MERRF)	Myopathy, blindness, deafness; elevated lactate; cardiomyopathy with Wolff–Parkinson–White (WPW) syndrome. mtDNA variant (*MT-TK* most common).
Neuropathy, ataxia and retinitis pigmentosa (NARP)	Late-childhood or adult onset; basal ganglia involvement. mtDNA variant (*MT-ATP6* most common).
Pearson syndrome	Sideroblastic anemia, pancreatic dysfunction. mtDNA deletion.
Progressive external ophthalmoplegia (PEO)	Ptosis, ophthalmoplegia and proximal limb weakness; presentation similar to KSS. mtDNA deletion.

CK: creatinine kinase.

**Table 2:** Neuromuscular disorders with known MH susceptibility based on genotypic variants associated with each condition. Note that additional characteristic genotypes not associated with MH exist but are not listed [13].

Neuromuscular Disorder	MH-Associated Variants
Central core disease	RyR1
Centronuclear myopathy	RyR1
Congenital fiber type disproportion	RyR1, CACNA1S
Congenital myopathy with cores and rods	RyR1
Idiopathic hyperCKemia	RyR1
King–Denborough syndrome	RyR1
Multiminicore myopathy	RyR1, CACNA1S
Native American myopathy	*STAC3*
Nemaline rod myopathy	RyR1
Periodic paralysis	RyR1, CACNA1S

MH: malignant hyperthermia, RyR1: ryanodine receptor, CACNA1S: RyR1-activating protein, CK: creatinine kinasa.

**Table 3:** Features of general and disease-specific perioperative complications for patients with neuromuscular disease.

Perioperative Complications in Patients with NMDs	
**General issues**	**Features**
Respiratory	Hypoxia, atelectasis
	Hypercarbia
	Pneumonia (aspiration)
	Respiratory failure
Cardiac	Arrhythmia
	Ventricular dysfunction
**Disease-specific issues**	**Features**
Malignant hyperthermia Associated with CCD, MmD, King–Denborough syndrome, other congenital myopathies	Hyperkalemia
	Muscle rigidity
	Acidosis
	Hyperthermia
Rhabdomyolysis More likely with muscular dystrophies	Hyperkalemia
	Acute kidney injury
Metabolic disturbance More likely with mitochondrial disease	Hypoglycemia
	Lactic acidosis
Propofol infusion syndrome More likely with mitochondrial disease	Lactic acidosis
	Rhabdomyolysis
	Lipidemia
	Bradycardia

NMD: neuromuscular disorder, CCD: central core disease, MmD: multiminicore disease.
